# Deleterious Mutations and the Rare Allele Burden on Rice Gene Expression

**DOI:** 10.1093/molbev/msac193

**Published:** 2022-09-08

**Authors:** Zoe Lye, Jae Young Choi, Michael D Purugganan

**Affiliations:** Center for Genomics and Systems Biology, New York University, New York, NY 10003; Center for Genomics and Systems Biology, New York University, New York, NY 10003; Center for Genomics and Systems Biology, New York University, New York, NY 10003; Center for Genomics and Systems Biology, New York University Abu Dhabi, Abu Dhabi, United Arab Emirates

**Keywords:** crop, rare variant, gene dysregulation, stabilizing selection

## Abstract

Deleterious genetic variation is maintained in populations at low frequencies. Under a model of stabilizing selection, rare (and presumably deleterious) genetic variants are associated with increase or decrease in gene expression from some intermediate optimum. We investigate this phenomenon in a population of largely *Oryza sativa* ssp. *indica* rice landraces under normal unstressed wet and stressful drought field conditions. We include single nucleotide polymorphisms, insertion/deletion mutations, and structural variants in our analysis and find a stronger association between rare variants and gene expression outliers under the stress condition. We also show an association of the strength of this rare variant effect with linkage, gene expression levels, network connectivity, local recombination rate, and fitness consequence scores, consistent with the stabilizing selection model of gene expression.

## Introduction

All populations contain some level of deleterious mutation (Ohta 1973; Huber et al. 2017; Gaut et al. 2018). Strongly deleterious variants are purged from populations due to the action of purifying selection, while mildly deleterious variants can remain in populations at low frequencies (Kimura 1983). There is great interest in understanding the levels and consequences of deleterious mutation loads, since this is an important factor in understanding various evolutionary phenomena, including the maintenance of breeding systems, evolution of sex, domestication, and even species range expansion (Keightley and Eyre-Walker 2000; Peischl et al. 2013; Wright et al. 2013; Gaut et al. 2018; Lozano et al. 2021; Samayoa et al. 2021). Understanding the nature of deleterious mutations can also aid in identifying human disease genes, as well as developing new approaches in plant breeding (Lohmueller 2014; Kono et al. 2016, 2018; Moyers et al. 2018; Wallace et al. 2018; Labroo et al. 2021).

Considerable effort has gone into predicting the relative deleteriousness of mutations. In coding sequences, one can estimate the functional effects of a coding mutation based on how the mutation affects protein structure (Ng and Henikoff 2003), while in non-coding sequences, deleteriousness is often estimated based on sequence conservation (Davydov et al. 2010; Kono et al. 2018). The rarity of polymorphisms in a population can also serve as a signal for deleteriousness, given that variants can be held at low frequency by selection and de novo mutations are generally weakly deleterious (Loewe and Hill 2010; Gibson 2012). Indeed, rare variants are associated with stronger phenotypic effects compared to common variants (Marouli et al. 2017; Bloom et al. 2019).

Because of their potentially deleterious nature, rare variants (those whose minor allele frequency in populations are <5%) are recognized as an important source of variation in gene expression. Recently, an analysis found that rare variants account for 25% of gene expression heritability, while another approach found 5% gene expression heritability explained by singleton polymorphisms (Glassberg et al. 2019; Hernandez et al. 2019). Large effect expression quantitative trait loci (eQTLs) are enriched for rare variants, and studies are beginning to quantify rare variant contribution to gene expression variation (Li et al. 2014; Bloom et al. 2019; Glassberg et al. 2019).

Genes that are prone to aberrant expression are also enriched for rare and private variants in regulatory regions (Zeng et al. 2015). Rare genetic variants are also associated with aberrant gene expression and expression outliers in both human and plant systems (Montgomery et al. 2011; Zeng et al. 2015; Zhao et al. 2016; Chiang et al. 2017; Li et al. 2017, 2021; Kremling et al. 2018; Richter et al. 2019; He et al. 2022). Unsurprisingly, rare variants in expression outlier genes are enriched for variant classes likely to impact expression, including structural variants (SVs), splice site mutations, and polymorphisms near the transcription start site (TSS) (Chiang et al. 2017; Li et al. 2017).

The association of rare genetic variants with outliers in gene expression supports the prevailing model of gene expression evolution by stabilizing selection (Glassberg et al. 2019). Under this model, gene expression is under selection for an optimum level, and deleterious variants are predicted to perturb expression away from this optimum (Bedford and Hartl 2009; Hodgins-Davis et al. 2015; Hill et al. 2021). Indeed, gene expression patterns are generally conserved across species, although not all gene classes are conserved to the same extent (Lemos et al. 2005).

The extent of stabilizing selection on gene expression, however, is uncertain; when using conventional eQTL approaches, the ability to identify an eQTL varies at different allele frequencies and low frequency eQTLs tend to have inflated effect sizes (Tung et al. 2015; Huang et al. 2018; Glassberg et al. 2019). Despite this, studies do report an association between low allele frequencies and stronger effect sizes as evidence of stabilizing selection on gene expression (Li et al. 2014); this is observed even after taking ascertainment bias and allele frequencies into account (Josephs et al. 2015; Brown and Kelly 2022). Glassberg et al. also reported a greater contribution of rare variants to allele-specific expression and finds fewer regulatory variants around dosage-sensitive genes (Glassberg et al. 2019). Moreover, the relationship between *cis-* and *trans*-regulatory variation supports stabilizing selection; *cis-* and *trans-*eQTLs tend to evolve commensurate mutations that maintain gene dosage balance over time (Signor and Nuzhdin 2018). Patterns of gene evolution also reflect the importance of gene expression conservation for particular gene classes, as non-duplicated genes are less tolerant to regulatory mutations and older genes have fewer associated eQTLs (Keane et al. 2014; Popadin et al. 2014).

The stabilizing selection model for gene expression variation makes other predictions on the strength of the effects of rare sequence variants on gene expression at functional loci, but these have remained unexplored. For example, it would be expected that selection against deleterious mutations would be stronger in highly expressed genes as well as genes with high network connectivity (Garcia-Alonso et al. 2014; Kremling et al. 2018; Hämälä and Tiffin 2020). These loci would thus be expected to be depleted of deleterious mutations and display a weaker effect of rare variants on gene expression variation. This should also the case for genes in regions of high recombination, which are expected to have reduced numbers of rare deleterious mutaions due to the Hill–Robertson effect (Hill and Robertson 1966; Comeron et al. 2008). Finally, one would expect that mutations with stronger effects on fitness (Gulko et al. 2015; Joly-Lopez et al. 2020) would be more likely to impact gene expression variation.

In this study we test these key predictions of the stabilizing selection model, as well as generally characterize the role of rare variants in gene expression in rice (*Oryza sativa*). Rice is a critical crop providing the main food source for >50% of the world population (Wing et al. 2018). Rice has a complex demographic history, and during domestication rice populations underwent a series of introgressions and population bottlenecks (Choi et al. 2017; Liu et al. 2017; Wing et al. 2018). As with many domesticated species, rice has a higher proportion of deleterious genetic variation compared to its wild relative (Liu et al. 2017; Kou et al. 2020). Rice also transitioned from an outbreeding to a selfing reproductive mode, which rapidly exposes the deleterious effects of rare recessive mutations while allowing slightly/moderately deleterious variants to accumulate (Arunkumar et al. 2015).

We examine the relationship between rare genetic variants and outliers in gene expression in a population of largely *O. sativa* ssp*. indica* rice landraces, examining the effects of multiple variant classes (single nucleotide polymorphisms [SNPs], insertion/deletion mutations [indels], and SVs). We look at the influence of gene expression level, recombination rate, and gene expression connectivity on the association between rare sequence polymorphisms and extremes in gene expression, and probe this both under normal unstressed wet and stressful dry (drought) field environments. Additionally, we show that variants that have a higher probability of having a fitness consequence have stronger associations with outliers in gene expression (Joly-Lopez et al. 2020).

## Results and Discussion

### Rare Genetic Variants in Rice

We identified rare genetic variants (<5% minor allele frequency) in a population of 129 *O. sativa* landraces, which included 105 cultivars of *O. sativa* ssp*. indica* and 24 of the closely related circum-*aus* variety group (supplementary table S1, Supplementary Material online) using whole genome re-sequencing data (Groen et al. 2020). We included SNPs, small indels (<20 bps in length), and SVs (>20 bps in length) in this analysis; unresolved genomic breakpoints were also categorized as SVs. Across the rice genome of ∼400 Mb in length, we initially identified 9,762,370 SNPs, 1,968,501 indels and 110,541 SVs, of which 484,340 SNPs, 294,929 indels and 18,509 SVs across the entire population had <5% frequency. On average there were 57,345 rare variants discovered in each genotype, the majority of which are found in noncoding sequences.

### Extremes of Gene Expression Is Associated With Accumulation of Rare Genetic Variation

**Fig. 1.**
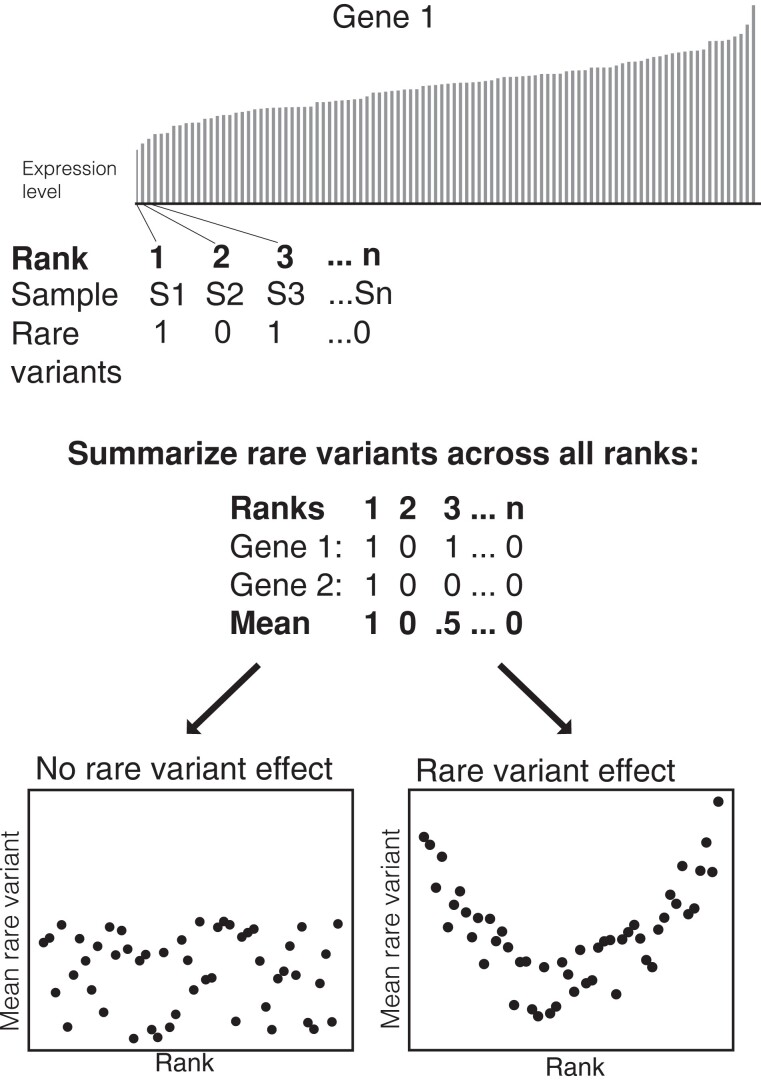
Schematic showing the calculation of mean rare variants per rank. The expression level for each gene is ranked, and the number of rare variants for a given individual at a given rank is counted and summarized across all genes. If there is a relationship between rare variants, a U-shaped plot of mean rare variants vs. ranks is expected.

Under the stabilizing selection model, deleterious variants that affect gene expression, which are likely rare, would tend to increase or decrease gene expression levels away from the intermediate optimum level (Zhao et al. 2016). We therefore expect to see an increase in the number of rare sequence variants in *cis*-regions of a gene within rice varieties that display extremes of gene expression in a population. To test this prediction, we examined the relationship between rare genetic variation and levels of gene expression in our rice population, using a rank-based approach (Zhao et al. 2016; Kremling et al. 2018). In this analysis, samples are ranked by gene expression level for each gene in the transcriptome, and the number of rare variants for each individual at that gene is counted (fig. 1). We then calculate the mean number of rare variants at each rank position across all genes (fig. 1). Our analysis included rare variants occurring in the gene coding region and 2 kb upstream of the TSS.

For gene expression data, we used 3′ mRNA expression data from rice leaves for our sample population collected under both normal wet paddy and dry (drought) field conditions (Groen et al. 2020). The 3′-mRNA-Seq assay was done in triplicate on 50-day old leaves; in the dry conditions, the plants were subjected to water deprivation starting 30 days after planting (Groen et al. 2020). We limited our analysis to genes that are robustly expressed (i.e., with non-zero expression levels in >85% of the varieties). This threshold limited our analysis to 4,046 genes in the wet condition and 3,508 genes in the drought conditions; 3,340 expressed genes overlapped between these two field environments. We were able to identify rare variants within the coding region and 2 kb upstream of these robustly expressed genes, including 65,913 SNPs, 12,602 indels, and 5,898 SVs.

**Fig. 2.**
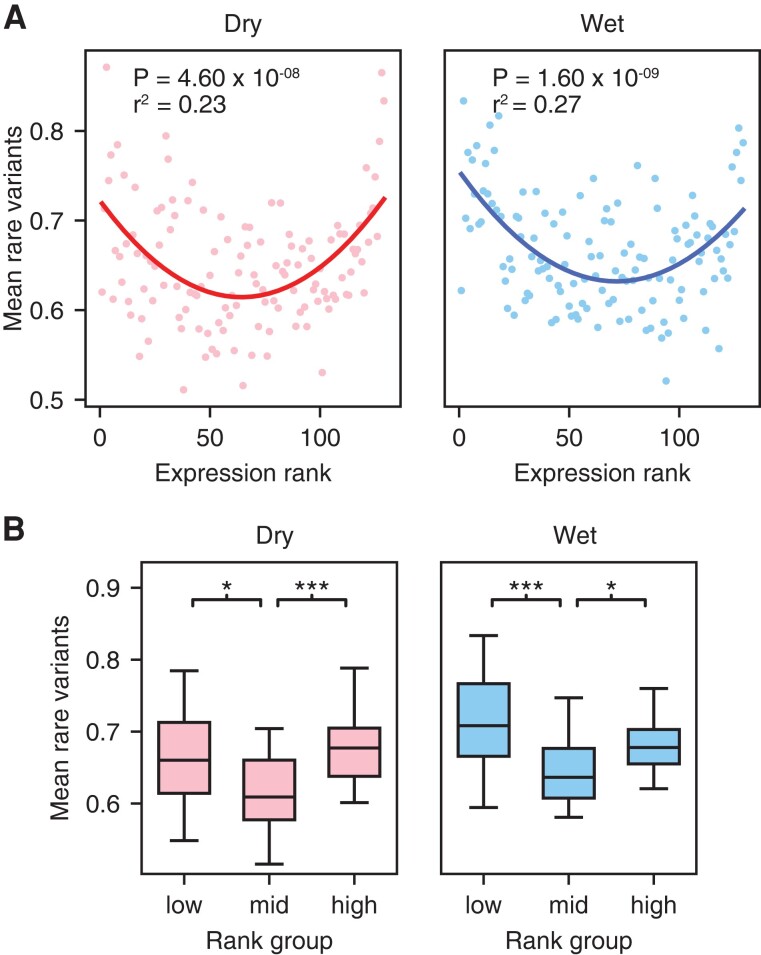
The rare variant effect on rice gene expression. (*A*) Mean rare variants per expression rank under wet and droughts stress (dry) conditions described by a quadratic curve. (*B*) Box plots of mean rare variants per expression rank. For low ranks (bottom 20%) middle ranks (40–60%), and high ranks (top 20%). Mann–Whitney comparison of distributions (* *P* < 0.05; *** *P* < 0.0005).

We found an association between rare genetic variation and outlier gene expression in rice under both wet and dry conditions. As in previous studies, this manifests itself as an excess of rare variants at the extreme high and low ranks of genes expression, and the pattern fits a quadratic curve (wet, *r*^2^ = 0.27, *P* < 1.6 × 10^−9^; dry, *r*^2^ = 0.24, *P* < 4.6 × 10^−8^) (fig. 2*A*) (Zhao et al. 2016; Kremling et al. 2018).

While the above analysis indicates a fit of the data to a quadratic curve (Zhao et al. 2016), this may not necessarily reflect significant rare variant enrichment associated with outlier gene expression. We therefore sought other ways to test the association of rare variants with outlier gene expression. As an alternative way to quantify the magnitude of the burden of rare variants, we tested the direct hypothesis that the extreme ranks in gene expression indeed have more rare variants compared to the middle ranks. We compared the mean number of rare variants per expression rank for the highest and lowest 20% of the ranks to the middle (those in the upper 40 to 60% of the ranks in gene expression) (fig. 2*B*). These were all significant using a Mann–Whitney comparison of mean rare variants per rank. Under wet conditions, comparison of the high vs. middle ranks had *P* = 2.62 × 10^−4^ and low vs. middle ranks *P* = 9.25 × 10^−3^, while under dry conditions, comparison of the high vs. middle ranks had *P* = 1 × 10^−4^, and low vs. middle ranks *P* = 0.005. We describe the association of rare variants with outliers in gene expression ‘the rare variant effect;’ this pattern is a manifestation of the deleterious burden of rare alleles that can contribute to a reduction of fitness.

## Rare Allele Burden in Normal vs. Stress Environmental Conditions

Under a model of stabilizing selection, gene expression becomes canalized whereby the effects of genetic variation on gene expression is buffered to minimize variation (Gibson and Wagner 2000; Gibson and Dworkin 2004). Gene regulatory networks contain buffering motifs and redundancy that promote robustness throughout the network (MacNeil and Walhout 2011; Siegal and Leu 2014). The ensuing canalization of gene expression patterns could lead to the accumulation of cryptic genetic variation (Gibson and Dworkin 2004; Paaby and Rockman 2014). Under the model of canalization, traits are buffered up to a certain threshold, past which greater perturbations can lead to de-canalizing and the release of the cryptic genetic variation (Paaby and Rockman 2014). Accumulation of novel cryptic variants can lead to deleterious loads under stressful or novel conditions and the release of expression regulatory variation in response to temperature variation has been observed experimentally in *Drosophila* and *C. elegans* (Li et al. 2006; Chen et al. 2015; Snoek et al. 2017).

Consistent with this, we observe an increase in the number of rare variants under stress conditions that may be associated with cryptic genetic variation. In indica rice, the rare variant effect on gene expression appears to differ between environments, and the effect is more pronounced under the drought stress conditions. To examine this difference between conditions, we devise the parameter φ = E_r_/M_r_, which is the ratio of the total number of rare variants at the extreme ranks (E_r_ = number of rare variants in the highest 10% and lowest 10% expression ranks) vs. the middle ranks (M_r_ = number of rare variants between the 40 and 60% expression ranks). The ratio φ = E_r_/M_r_ = 1.158 in drought stress conditions is ∼4.5% higher than in normal wet paddy conditions (φ = 1.108) (supplementary table S2, Supplementary Material online). This increase in φ in the stressful dry conditions is small and not significant in a permutation test (permuted 1,000 times, *P* < 0.15), but nevertheless the elevated number of rare variants in extremes vs. middle ranks of gene expression under stress conditions is significant in a contingency test (Fishers exact test *P* = 1.6 × 10^−8^, log odds ratio = 1.39; see supplementary table S2, Supplementary Material online for rare variant counts). The pattern remains significant when only examining genes shared between the two conditions (Fishers exact *P* = 0.0067, log odds ratio = 1.02). This suggests that more deleterious mutations are associated with gene expression outliers under environmental stress conditions.

### Rare Variant Effect Among Different Variant Types

We test whether different classes of genetic variants may have different levels of association with expression outliers. SVs, for example, create larger mutational lesions in the genome than SNPs or indels, and are generally associated with greater effects on expression phenotypes, (Han et al. 2020; Jakubosky et al. 2020), and are enriched in regions near genes with outlier expression (Chiang et al. 2017). We thus expect SVs to show a more pronounced rare variant effect on gene expression.

**Fig. 3.**
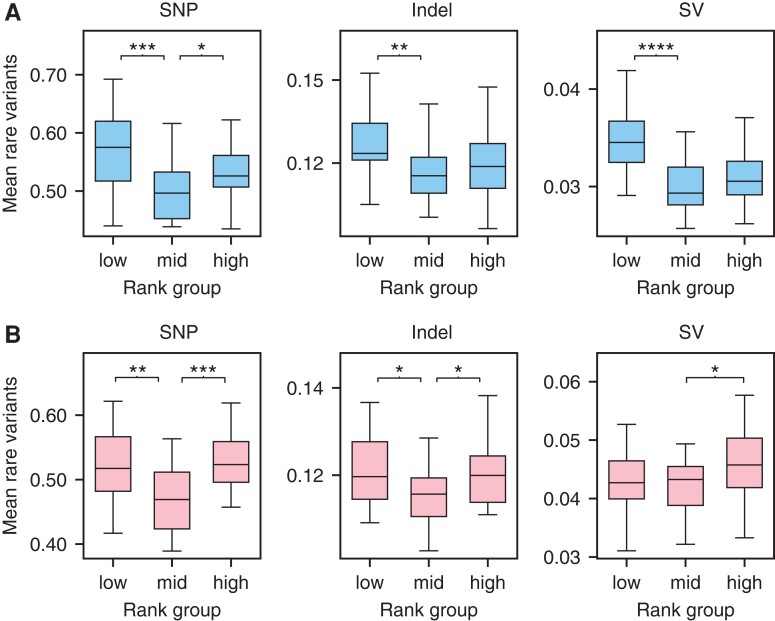
Box plots of mean rare variants per expression rank for SNPs, indels, and SVs. (*A*) Wet condition (*B*) dry condition. For low ranks (bottom 20%) middle ranks (40–60%) and high ranks (top 20%). Mann–Whitney comparison of distributions (*P < 0.05; **P < 0.005; ***P < 0.0005; ****P < 0.00005).

We conducted our analysis on each variant class independently and demonstrated that each class contributes to the rare variant effect (fig. 3). For SNPs, which comprise the largest variant class, we observe an increase in the number of rare variants at extreme ranks in both wet and dry conditions (supplementary table S2, Supplementary Material online). Interestingly, under wet condition there is an increase in rare indel and SV variants (Mann–Whitney, SV *P* = 1.48 × 10^−5^; indel *P* = 0.0018) that appear to lead to decreased gene expression (fig. 3*A*). This bias for lower expression is expected given that SVs (and possibly indels) are more likely to lead to loss-of-function mutations that decrease gene expression (Conrad and Hurles 2007). Interestingly, however, the opposite pattern is observed under dry conditions, at least for SVs (Mann–Whitney, *P* = 0.02) (fig. 3*B*), and it is unclear why this pattern is reversed under stress conditions. It should be noted that since we observe opposite trends in wet vs., dry conditions, we do not believe that this environment-specific skew in rare variant enrichment occurs because of bias associated with mapping 3′mRNA sequencing reads to the reference genome.

### The Effect of Linkage

**Fig. 4.**
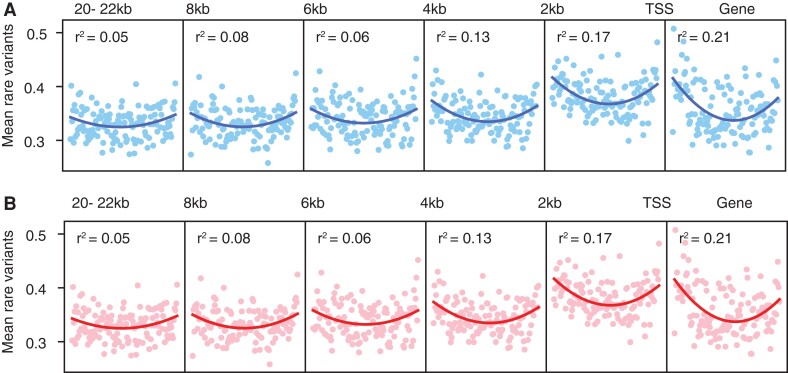
Mean rare variants per expression rank and linkage. Mean rare variants per expression rank within genes and in 2 kb regions upstream of the TSS for wet (*A*) and dry (*B*) conditions. *r*^2^ values are from the best-fitted quadratic model.

We compared the rare allele burden at different positions relative to the gene TSS, with the expectation that the rare variant effect would be weaker in regions further away from the TSS. We find the rare variant effect most pronounced in the gene body and in the 2-kb genomic region immediately upstream of the TSS (fig. 4*A*). This is observed in the quadratic fit of the data in wet conditions, which is greatest in the gene body (*r*^2^ = 0.21) and the 2 kb region upstream of the TSS (0–2 kb window, *r*^2^ = 0.17 vs. 2–4 kb window, *r*^2^ = 0.13). The effect declines in genomic regions further upstream from the TSS, where the quadratic fit is markedly reduced, with the lowest value in the most distal region (>20 kb upstream of the TSS) (fig. 4*A*; supplementary table S3, Supplementary Material online). We find a similar pattern in dry conditions (fig. 4*B*; supplementary table S3, Supplementary Material online).

**Fig. 5.**
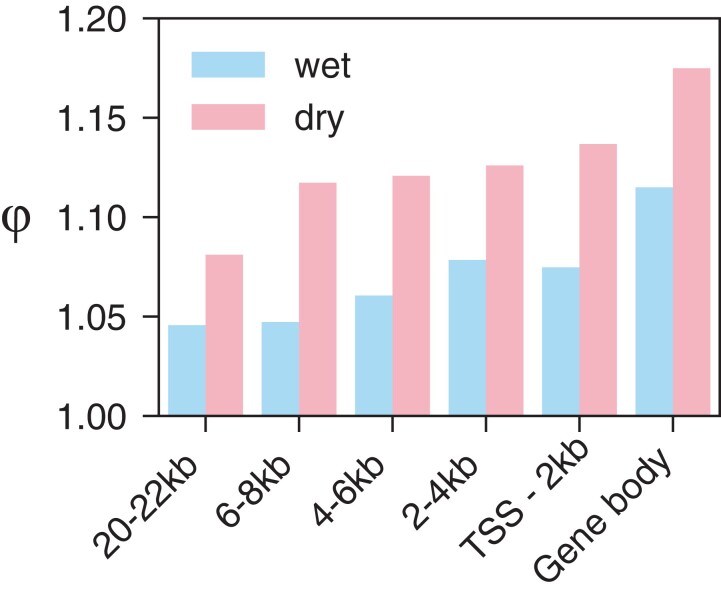
φ for genic regions and windows upstream of the gene in wet (blue) and dry (red) conditions. Wet corresponds to the left bar and dry corresponds to the right bar.

This linkage effect is also observed with the parameter φ = E_r_/M_r_. In both wet and dry conditions, this parameter is high (wet φ = 1.07, dry φ = 1.14) in the 2-kb region directly upstream of the TSS, which is consistent with the presence of regulatory sequences in the gene promoter (fig. 5). The increase in the proportion of rare variants associated with the extremes of gene expression between the 2-kb region upstream of genes vs. unlinked sequences (>20 kb regions) is small—2.8% in wet and 5.2% in dry conditions. Nevertheless, there is a clear trend of decreasing φ with increasing distance from the gene body (see fig. 5). These results are consistent with rare variants having a greater impact in gene expression the more proximal they are to the gene and its promoter sequence (Li et al. 2017); it is the linked sites that are likely to be functionally important in a gene’s expression.

Interestingly, the parameter φ is also high (e.g., wet φ = 1.11) in the coding sequence of the gene, which is consistent with other studies that have found that rare variants from genic regions are also associated with outlier gene expression (Chiang et al. 2017; Li et al. 2017; Han et al. 2020). This could partially be explained by linkage between the gene body and causal rare variants in proximal regulatory regions, or possibly other regulatory elements within a gene that affects transcript levels. Decreased mRNA levels, for example, may arise through loss-of-function mutations in coding regions that lead to nonsense mediated decay (Karousis and Mühlemann 2019). Variants in the coding sequence could also influence expression if a gene has auto-regulatory functionality or by triggering changes in feedback loops controlling that gene’s expression (Rockman and Kruglyak 2006). Expression stimulation by introns is another well characterized source of expression regulation in many plant species (Rose 2019).

### Deleterious Rare Variation and Recombination Rates

The Hill-Robertson effect predicts that selection is less effective in regions of reduced recombination, and thus deleterious variants are more likely to accumulate in regions of low recombination (Hill and Robertson 1966; Comeron et al. 2008). Enrichment of deleterious variants in low recombining regions is observed in rice and many other systems (Charlesworth and Campos 2014; Renaut and Rieseberg 2015; Rodgers-Melnick et al. 2015; Liu et al. 2017; Kono et al. 2019; Kim et al. 2021). We therefore expect that rare variants in low recombining regions should contribute more to the burden of rare alleles.

**Fig. 6.**
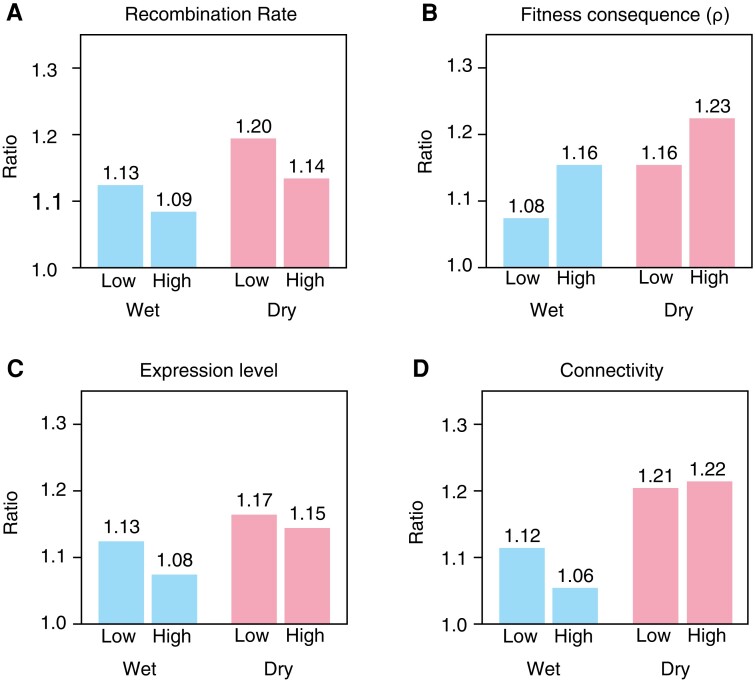
Effects of different factors on φ in wet (blue) and dry (red) environments. (*A*) Comparison of φ between gene groups divided into low and high recombining genes. (*B*) Comparison of φ calculated for SNPs with low and high fitness consequence scores. (*C*) Comparison of φ for low and high expression genes. (*D*) Comparison of φ between low and high connectivity genes.

We assigned recombination rates to each gene in our analysis based on a genetic map derived from a *O. sativa* japonica/aus cross and then classified the genes into high and low recombination rate genes based on the top and bottom 50th percentile (Harushima et al. 1998). The ratio φ = E_r_/M_r_ is higher in low recombination genes (φ = 1.13) compared to high recombination genes (φ = 1.09) in wet conditions; the same increase is seen in dry conditions (fig. 6*A*; supplementary table S2, Supplementary Material online). There is also enrichment of rare variants in the extremes of gene expression for the low recombining compared to the high recombining genes; the increase in low recombining regions is 3.7% in wet conditions and 5.3% in dry conditions and these are significant (Fishers exact, wet: *P* = 3.77 × 10^−5^, log odds = 1.04; dry: *P* = 2.7 × 10^−5^, log odds = 1.05; supplementary table S2, Supplementary Material online). Our observation of a reduced rare variant effect in genes in high recombination regions demonstrates the role of purifying selection on gene expression levels, which reduces the levels of deleterious mutations as predicted by the Hill–Robertson effect.

### SNPs With Different Fitness Consequence Scores

The relative fitness effects of mutations are often estimated by modeling sequence conservation across species (Joly-Lopez et al. 2016). It has been observed that gene expression outlier variants are associated with conserved sequences in human tissues (Li et al. 2017; Richter et al. 2019). Recently, models have been developed that incorporate not only macroevolutionary sequence variation, but within- and between-species genetic variation to model the effects of selection on specific genomic sequence features (Gronau et al. 2013; Gulko et al. 2015; Joly-Lopez et al. 2020). Such models lead to the inference of fitness consequence (fitCons) maps for a species, and one was recently developed for rice (Joly-Lopez et al. 2020). In these maps, a fitness consequence score (ρ) is assigned to different positions in the genome; ρ ranges from 0 to 1 representing the probability that a mutation at a specific site has a fitness consequence, and is thus a measure of selection acting on a genomic region (Joly-Lopez et al. 2020).

For our analysis, we used the rice fitness consequence map to assign a ρ value for each rare SNP in our dataset. Based on the distribution of ρ across the rice genome, we then classified SNPs into high (ρ > 0.2) and low (ρ < 0.1) ρ classes; the thresholds were determined by the distribution of fitness consequence scores across the rice genome (Joly-Lopez et al. 2020). We expected the rare variant effect to be more pronounced with SNPs in the high ρ class, since mutations in this class are expected to be more deleterious (i.e., with a greater effect on fitness). As predicted, the high ρ rare SNPs produced a significantly stronger rare variant effect compared to the low ρ rare SNPs in both wet (∼7.4% increase) and dry (∼6.03%) conditions (Fishers exact, wet: *P* = 2.04 × 10^−7^, log odds = 1.07; dry: *P* = 3.86 × 10^−5^, log odds = 1.06) (fig. 6*B*; supplementary table S2, Supplementary Material online).

### Highly Expressed Genes Have Lower Burden of Rare Alleles

pt?>Highly expressed genes are under greater purifying selection (Lemos et al. 2005; Larracuente et al. 2008; Gout et al. 2010). There are two possible scenarios on how the rare variant effect can manifest itself under this increased selection. First, increased purifying selelction could lead to a more pronounced rare variant effect by increasing the number of deleterious variants in a population. Alternatively, very strong purifying selection can purge deleterious alleles, leading to less deleterious variants in the population and thus a weaker rare variant effect. Interestingly, we find that the rare variant effect is indeed weaker among highly expressed genes compared to those expressed at lower levels, supporting the second scenario. We split our gene dataset into the highest and lowest 50th percentile of gene expression levels. Similar to what has been shown in maize (Kremling et al. 2018), we find that the quadratic curve is more pronounced in the lower expressed genes.

This pattern can also be seen in a significant enrichment in the number of rare variants in the extremes among lower expressed genes (wet φ = 1.13; dry φ = 1.17) compared to genes with higher expression (wet φ = 1.08; dry φ = 1.15) (Fishers exact, wet: *P* = 5.4 × 10^−5^, log odds = 1.04; dry: *P* = 0.05, log odds = 1.02) (fig. 6*C*; supplementary table S2, Supplementary Material online) (Kremling et al. 2018). Correlation between expression level and recombination has been observed in other studies; however, we found no correlation between recombination rate and gene expression that might explain this pattern (wet: Pearson r = −0.02, *P* = 0.33; dry: r = −0.01, *P* = 0.37) (Larracuente et al. 2008). These results suggest that purifying selection may act with greater strength in highly expressed genes, and is weaker in genes of lower expression such that the latter harbors more deleterious mutations.

### The Impact of Network Connectivity

Stabilizing selection on gene expression should be affected by the properties of the network of interactions of a given gene. We obtained a measure of connectivity for each gene in our data set from a field study of rice (Plessis et al. 2015); our measure describes how strongly a gene’s expression correlates with the mean expression of genes that belong to a co-expressed cluster. We estimated connectivity for 2,936 robustly expressed genes in the wet condition and 2,568 genes in the dry condition (Plessis et al. 2015). We then classified genes into high connectivity and low connectivity groups (high connectivity genes: *r* = 0.80–0.99; low connectivity genes: *r* = 0.16–0.77) and examined the rare variant effect.

In wet conditions, the rare variant effect was stronger in low connectivity genes, and the number of rare variants in expression outliers was lower among high connectivity genes (high connectivity φ = 1.06; low connectivity φ = 1.12; Fishers exact *P* = 5 x10^−5^, log odds = 1.05) (fig. 6*D*; supplementary table S2, Supplementary Material online). In the dry condition, the rare variant effect is similar in both high and low connectivity genes (high connectivity φ = 1.22, low connectivity φ = 1.21, Fishers exact *P* = 0.71, log odds = 0.99), and there is no significant difference.

Gene connectivity is correlated with expression level; therefore it is difficult to disentangle the relationship between rare variants and gene connectivity vs. that of gene expression (Williamson et al. 2014; Josephs et al. 2017). However, Brown et al, used a similar measure of co-expression module connectivity and non-expression correlated genes, and showed that eQTLs in highly connected genes occur at lower population frequencies than low connectivity genes (Brown and Kelly 2022). Nucleotide interactome analysis also found that deleterious variants are more frequent at the periphery of the interactome under normal conditions, while under a disease state deleterious variants were present in more central nodes (Garcia-Alonso et al. 2014). Our results are consistent with these studies, at least in the normal wet conditions. Together, our results suggest that deleterious mutations are at lower levels in highly connected genes, where such mutations may have greater pleiotropic consequences and are therefore more likely to have been purged by purifying selection.

## Summary

We have investigated the effects of rare genetic variants on gene expression in the key domesticated crop *O. sativa*. Our work is consistent with results of similar rank-based analysis of the effects of rare variants on gene expression in humans and maize (Zhao et al. 2016; Kremling et al. 2018), and additionally shows the contributions of different types of genetic variants to expression variation. The effects of deleterious variation on fitness are particularly relevant in domesticated crops, which have undergone population bottlenecks during their evolution, and more so in a selfing species such as rice. As also observed in humans and maize, the ability to conduct large-scale gene expression assays permits us to observe the effects of rare deleterious expression on thousands of gene expression phenotypes.

Our results demonstrate how the burden of rare variants varies among genes based on attributes such as recombination rate, expression level, and connectivity, all of which support a stabilizing selection model for gene expression; the pattern is summarized in Table 1. We find, for example, that the rare variant effect is weaker among highly recombining regions, demonstrating the role of recombination in removing deleterious variants from the population. Differences in the rare variant effect among different classes of genes also reflect that not all genes are under equal levels of stabilizing selection, and the relative tolerance of different gene classes to aberrant gene expression could provide insights into the relative contributions of molecular evolution through regulatory and coding sequence change. Finally, our study examines the relationship between rare variants and outliers in gene expression under multiple environmental conditions. Analyzing environmentally induced changes in gene expression is a unique way to survey the effects of cryptic variation simultaneously across a large number of traits. The transcriptome response to stress is both specific and non-specific, and the increased dysregulation associated with deleterious variants under stress could be due to a general stress response or reflect environment-specific behavior (López-Maury et al. 2008).

**Table 1.** Summary of Patterns of Rare Variant Effect.

	Strong effect	Weak effect
Linkage	Linked	Unlinked
Recombination rate	Low recombination	High recombination
Environment	Dry/stress	Wet/normal
Expression level	Lower expression	Higher expression
Fitness consequence	High **ρ**	Low **ρ**
Connectivity	Low connectivity	High connectivity

Studying the extent and nature of deleterious mutations is important in understanding their role in evolutionary phenomena (Keightley and Eyre-Walker 2000; Gibson 2012; Wright et al. 2013; Gaut et al. 2018). There are also more immediate practical reasons, as it has been suggested that domesticated crop species contain a large mutational burden that could restrain agricultural yields (Ramu et al. 2017; Yang et al. 2017; Moyers et al. 2018; Wallace et al. 2018). The reduction of this deleterious mutational burden is already a goal of many breeding efforts (Wallace et al. 2018; Labroo et al. 2021), and dissecting the effects of these mutations in gene expression can provide new avenues of investigation and help advance future crop breeding.

## Materials and Methods

### Gene Expression Data

Whole genome re-sequencing data and sequencing of 3′ mRNA tags for 129 indica samples are part of a previously published dataset (Groen et al. 2020). The RNA 3′ reads were processed using the Drop-seq pipeline (https://github.com/broadinstitute/Drop-seq/tree/master/src/scripts) and the STAR aligner (version 2.5.2b) (https://github.com/alexdobin/STAR). In summary, the Shuhui498 (R498) reference genomeV3 (http://mbkbase.org/R498/) was prepared as the reference using STAR genomeGenerate and then fastq reads are converted to unaligned BAM files using picard FastqtoSam for each library. Drop-seq_alignment.sh from the Drop-seq pipeline (Drop-seq_tools version 1.12) was run for each BAM file, and the results are converted to digital expression files using “DigitalExpression” from the Drop-seq package. Digital expression files were merged and read count normalized as described in Groen et al., 2020. All downstream analysis was carried out on log2(normalized transcripts-per-million value + 1).

### SNP/Indel Calling

Raw FASTQ reads were downloaded from SRA BioProject accession numbers PRJNA422249 and PRJNA557122 (Gutaker et al. 2020). SNP/indel calling was performed using GATK v.4.0.1.2 implemented in a Nextflow pipeline (https://github.com/zlye/RVE). In summary, reads were mapped against Shuhui498 reference genome (Du et al. 2017) using the global aligner BWA-MEM v.0.7.01 mode (Li and Durbin 2009). FASTQ sequences from the same samples were merged and duplicate reads are removed using Picard MarkDuplicates to generate sam files (http://broadinstitute.github.io/picard/). SAM files were validated and indexed to make bam files for each sample which were used to call haplotypes with gatk-4.0.1.2 HAPLOTYPE CALLER. gVCF haplotypes were joined using gatk-4.0.1.2 GenomicsDBIImport and then called across the population using gatk-4.0.1.2 GenotypeGVCFs to produce a set of raw indels and SNPs. SNPs and indels were filtered for biallelic variants. SNPs and indels were also filtered for quality based on normalized by depth (QD), mapping quality (MQ), MappingQualityRankSumTest (MQRankSum), read strand bias from strand odds ratio (SOR) read position bias from Wilcoxon’s test (ReadPosRankSum) and strand bias from Fisher’s test (FS). The following filters were applied to SNPs: QD > 2, FS < 60, MQ > 40, SOR < 4, MQRankSum > −12.5; ReadPosRankSum > −8. The following filters were applied to indels: QD > 2, FS < 200, SOR < 10. Indels were filtered for variants 20 bps or less in length.

### SV Calling

SVs were discovered from BAM files using GRIDSS v 2.8.0 (Cameron et al. 2017). Samples with multiple sequencing libraries were jointly processed by GRIDSS. Deletions, duplications, insertions, and inversions were resolved from the breakends (BND) discovered by GRIDSS using custom scripts (https://github.com/zlye/RVE). Deletion and duplication variants were annotated for read-depth using *Duphold* v0.12 (Pedersen and Quinlan 2019). SVs were filtered for deletions with read-depth fold-change relative to flanking regions < 70%, and for duplications with fold-change relative to bins in the genome with similar GC-content > 130%. BNDs, insertions, and inversions were retained.

Although SVs are commonly categorized as variants >50 base pairs, we included variants greater than 20 base pairs because GRIDSS performs well in identifying variants in this size range and we sought to maximize the inclusion of genetic variants in our dataset (Cameron et al. 2017; Kosugi et al. 2019). We discarded SVs >200 kb as probably false positives. SVs were merged into a population dataset using a custom python scrip (https://github.com/zlye/RVE). Merging required 50% reciprocal overlap and breakends to be within 1 kb of each other to join SVs across samples. Exact genomic position match was required to merge BNDs. SVs with overlapping coordinates within an individual could represent complex rearrangements and cause ambiguity when merging across samples; thus, these were excluded.

### Rare Variant and Expression Filtering

SNPs and indels were filtered for <5% frequency in the population and a minimum of at least one homozygous individual or three heterozygous individuals. We chose this filtering scheme to account for the possibility of sequencing error in singleton heterozygotes. SVs were also filtered for frequency <5% in the population. Singletons were permitted among SVs because SV calls are derived from multiple signals in sequencing data—split-reads, read-depth, and assembly; thus, the probability of false positive singleton SVs is much lower than SNPs or indels.

Genes are considered in the analysis if they are expressed in two replicates in at least 85% (109/129) individuals. Genes with >50% reciprocal overlap with predicted transposable elements were removed from the data set (15 genes were removed). Gene expression is calculated as the mean expression across the three replicates.

### Fitness Consequence Score Analysis

The rice fitness consequence map (Joly-Lopez et al. 2020) is anchored on the rice japonica Nipponbare reference Os-Nipponbare-Reference-IRGSP-1.0 (Sasaki and Burr 2000), which can be downloaded from RAP-DB (https://rapdb.dna.affrc.go.jp/). Nipponbare coordinates were determined for each rare SNP using LiftOver from the UCSC genome browser (Kent et al. 2002). Fitness consequence map was downloaded from the fitcons browser (http://purugganan-genomebrowser.bio.nyu.edu/insightJuly2018/greenInsight.html) and intersected with the rare SNP Nipponbare coordinates to assign fitcons ρ scores to each rare SNP.

### Connectivity

We obtained measures of expression connectivity based on gene co-expression modules derived from transcriptome data of 240 rice samples under wet and dry conditions from Plessis et al. (Plessis et al. 2015). Gene connectivity was defined as the correlation with the mean of gene expression in the cluster, which we obtained for 3,071 genes in our dataset of robustly expressed genes.

### Statistical Analysis

All statistical analysis were carried out using Python package SciPy (Virtanen et al. 2020).

## Supplementary Material

msac193_Supplementary_Data

## Data Availability

Gene expression matrixes, SNP, INDEL and SV data sets, and summary data are available on Zenodo (https://zenodo.org/) with DOI 10.5281/zenodo.6812091
